# Case report: Functional characterization of a novel *CHD7* intronic variant in patients with CHARGE syndrome

**DOI:** 10.3389/fgene.2023.1082100

**Published:** 2023-02-09

**Authors:** Cesare Rossi, Sherin Ramadan, Cecilia Evangelisti, Simona Ferrari, Maria Accadia, Reha M. Toydemir, Emanuele Panza

**Affiliations:** ^1^ UO Genetica Medica, IRCCS Azienda Ospedaliero-Universitaria di Bologna, Bologna, Italy; ^2^ Dipartimento di Scienze Mediche e Chirurgiche, Università di Bologna, Bologna, Italy; ^3^ Servizio di Genetica Medica, Ospedale “Cardinale G. Panico”, Tricase (LE), Italy; ^4^ Department of Pediatrics, University of Utah, Salt Lake City, UT, United States

**Keywords:** CHD7, CHARGE syndrome, splice site mutation, minigene, intronic variant

## Abstract

**Background:** Because CHARGE syndrome is characterized by high clinical variability, molecular confirmation of the clinical diagnosis is of pivotal importance. Most patients have a pathogenic variant in the *CHD7* gene; however, variants are distributed throughout the gene and most cases are due to *de novo* mutations. Often, assessing the pathogenetic effect of a variant can be challenging, requiring the design of a unique assay for each specific case.

**Method:** Here we describe a new *CHD7* intronic variant, c.5607+17A>G, identified in two unrelated patients. In order to characterize the molecular effect of the variant, minigenes were constructed using exon trapping vectors.

**Results:** The experimental approach pinpoints the pathogenetic effect of the variant on *CHD7* gene splicing, subsequently confirmed using cDNA synthetized from RNA extracted from patient lymphocytes. Our results were further corroborated by the introduction of other substitutions at the same nucleotide position, showing that c.5607+17A>G specifically alters splicing possibly due to the generation of a recognition motif for the recruitment of a splicing effector.

**Conclusion:** Here we identify a novel pathogenetic variant affecting splicing, and we provide a detailed molecular characterization and possible functional explanation.

## Introduction

CHARGE syndrome (MIM 214800) is a rare autosomal dominant condition occurring in approximately one in 10,000 to 15,000 newborns (Hudson et al., 2017). The name CHARGE is an acronym referring to the cardinal features classically described in these patients: Coloboma, Heart defects, choanal Atresia, growth Retardation, Genital abnormalities and Ear abnormalities (Pagon et al., 1981). However, not all patients have all of these findings, and most patients present with additional features, resulting in a highly variable clinical phenotype. In addition, these clinical characteristics are common in many other genetic disorders. Therefore, differential diagnosis based on clinical presentation is challenging, and genetic testing is an essential component of the diagnostic workup (Corsten-Janssen et al., 2013).

Two genes have been associated with CHARGE syndrome: *SEMA3E* (MIM 608166) and *CHD7* (MIM 608892). The causative role of the former is uncertain: only a handful of CHARGE patients with *SEMA3E* variants have been reported since the initial publication by Lalani et al. (2004). In contrast, *CHD7* pathogenic variants are found in approximately 70% of patients (Vissers et al., 2004; Zentner et al., 2010).

*CHD7* is located on chromosome 8q12.1 and contains 38 exons. It encodes the Chromo-domain Helicase DNA-binding (CHD) protein 7, a 2,997 aminoacids protein belonging to a family of gene expression modifiers (Vissers et al., 2004). Seventy percent of reported *CHD7* mutations are non-sense or frameshift. While the remaining are splice site missense mutations (Zentner et al., 2010), and intragenic deletions spanning one or more exons. Hence, most of the mutations cause an early termination of protein synthesis, generating truncated proteins. This is in line with the hypothesis that *CHD7* haploinsufficiency is the pathogenetic mechanism that causes CHARGE syndrome (Sanlaville and Verloes, 2007; Bergman et al., 2011). This hypothesis is also supported by studies using knock-out mouse models, in which heterozygous animals recapitulate some of the peculiar features of CHARGE syndrome (Bosman et al., 2005; Hurd et al., 2007).

The genetic diagnosis of CHARGE syndrome remains challenging despite technological advances. Virtually all cases are sporadic due to *de novo* mutations (Bergman et al., 2011). Determining the pathogenicity of genetic alterations identified through clinical testing may be difficult, frequently leading to their classification as variants of unknown significance (VUS). Functional characterization of *CHD7* variants is therefore key to improving the molecular diagnosis of this condition.

In the present study, we investigated a novel intronic variant, c.5607+17A>G, found in two unrelated patients and originally classified as a VUS.

In order to demonstrate the pathogenicity of this novel intronic variant we combined bioinformatic predictions and functional analyses. Synthetic minigene constructs and *in vitro* analysis of the residual sample from one of the patients proved this variant to be pathogenetic at the level of RNA.

## Background

### Case report

**Patient 1.** The patient is male and was 7 years old at diagnosis. His clinical picture includes atresia of the choanae (subjected to surgery), hypogonadotropic hypogonadism and micropenis (under therapy with luteinizing hormone-releasing hormone analogue), bicuspid aortic valve, asymmetry of the buccal rhyme when crying and smiling due to hypoplasia of the left mouth corner depressor, motor impediment, and mild craniofacial dysmorphisms (slightly low implanted pinnae, prominent nasal root, hypoplastic nasal wings, mid-facial hypoplasia, high and narrow palate). He uses corrective lenses for myopia. ENT (ear, nose and throat) evaluation documented very slight bilateral conductive hearing loss. Re-evaluation of CT radiograms taken at birth showed “inner ear dysplasia”. The patient’s clinical features currently meet the 2005 Verloes criteria for the diagnosis of ‘incomplete CHARGE syndrome’, with two major criteria (choanal atresia and agenesis of the semicircular canals) and one minor criterion (hypogonadism).

**FIGURE 1:**
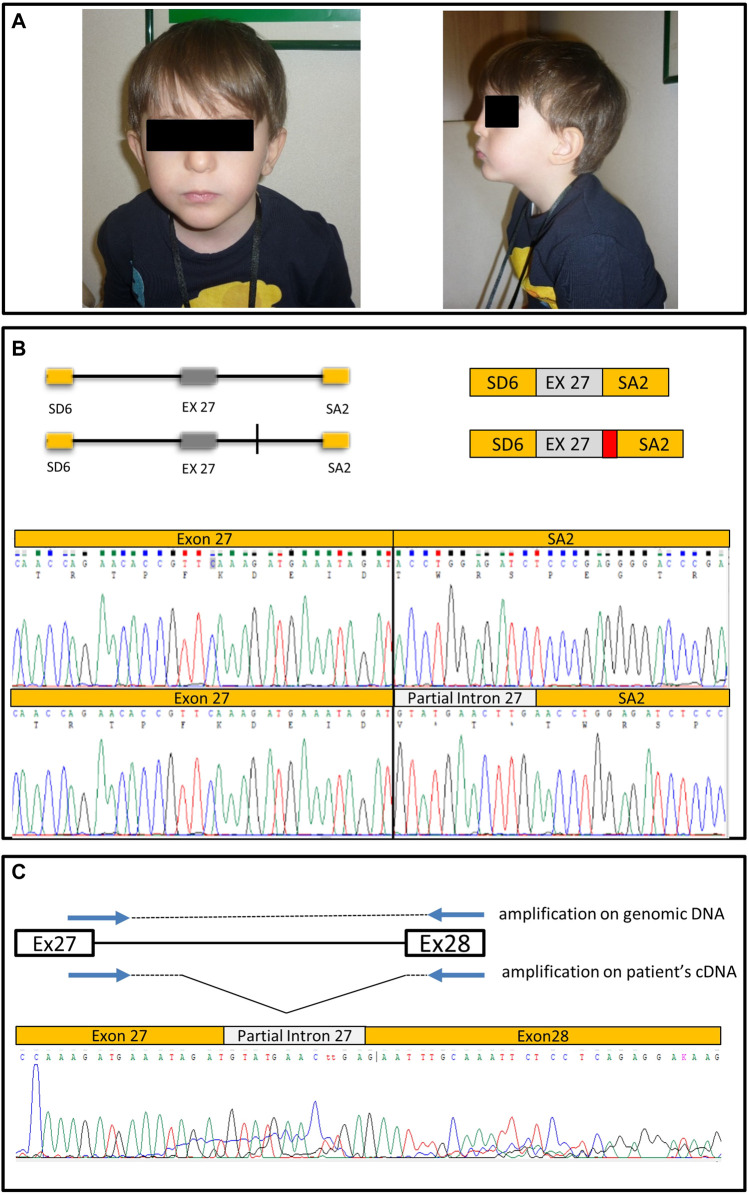
Clinical images, minigene structure and sequencing results. **(A)** Patient pictures. **(B)** Schematic representation of the wt genomic region cloned in pSPL3 corresponding to the portion of intron 26, exon 27 and intron 27 cloned in pSPL3, and of the construct with the A > G transition (vertical line). On the side is represented the effect on splicing of the wt construct and of the variant construct; in the latter case resulting in partial intron retention (in red) and a premature stop codon, as shown by the respective chromatograms. **(C)** Schematic of the strategy to amplify the pathologically spliced allele on patient’s cDNA, and chromatogram showing the retention of a part of intron 27.

All 38 *CHD7* coding exons, including at least 20 bases of exon/intron boundaries, were Sanger sequenced. A c.5607+17A>G heterozygous variant in intron 27 (NM_017780.4) was identified (Figure 1). Trio segregation analysis showed that the c.5607+17A>G variant occurred *de novo* in this patient.

**Patient 2.** This patient is also male, was 9-months old at diagnosis and presents four specific features of CHARGE (coloboma, cranial nerve dysfunction, middle ear ossification and outer ear malformation). He was G-tube dependent, and presented with swallowing problems, retrognathia, fluctuating conductive hearing loss in the right ear, sensorineural hearing loss in the left ear, and small bilateral optic disc colobomas. Renal ultrasound showed grade 1 pelviectasis. He also manifested a square, broad face with subtle frontal bossing, and cup-shaped ears. CT and MRI of the jaw including inner ear showed absence of the semicircular canal on left and partial absence on the right. SNP microarray analysis was normal. Sequencing *via* Sanger method identified the c.5607+17A>G intronic variant (Figure 1).

## Materials and methods

### Genetic analysis identifies a variant of unknown significance

All 38 *CHD7* coding exons were amplified by PCR, and purified products were Sanger sequenced. Purified sequence products were loaded on a ABI 3730 sequencer (Applied Biosystems) and electropherograms were analyzed by FinchTV (https://digitalworldbiology.com/FinchTV).

### Generation of constructs for minigene assay

An *in vitro* splicing assay was performed using an exon trapping vector. Wild-type (wt) and mutant constructs were generated from control genomic DNA by amplifying a region that includes intron 26, exon 27 and intron 27 of *CHD7*, and cloning the fragments into a pSPL3 vector (see Supplementary Material for a detailed description of the procedure).

### Nucleofection of HEK293 cells

The HEK293 human cell line was cultured in Dulbecco’s Modified Eagle’s Medium (DMEM, high glucose, with stable glutamine and sodium pyruvate, Euroclone) containing 10% fetal bovine serum, 1% penicillin/streptomycin and 1% non-essential amino acid mix, at 37°C in 5% CO_2_ incubator. One microgram of pSPL3 vectors, were electroporated into one million HEK293 cells, in buffer from kit V. Nucleofection was performed with an AMAXA Nucleofector (Lonza), using program A024. After being pulsed, recovered cells were incubated in culture medium for 72 h, and then analyzed.

### RNA extraction and cDNA synthesis

Total RNA was extracted from transfected cells using the RNeasy mini kit (Qiagen). Approximately 500 ng of total RNA was reverse transcribed with the RevertAid First Strand cDNA Synthesis kit, using random hexamers to synthesize cDNA, following manufacturer’s recommendations (Thermo Fischer). cDNA was amplified using pSPL3 vector-specific primers SD6 (5′-TCT​GAG​TCA​CCT​GGA​CAA​CC-3′) and SA2 (5′-ATC​TCA​GTG​GTA​TTT​GTG​AGC-3′). The size of amplified products was determined by electrophoresis in 2% agarose gel, stained with Midori staining dye (Elpis Biotech, Daejeon, Korea). PCR products were then purified (PCR and gel purification kit, Gene All), and sequenced (ABI 3730 Instrument, Applied Biosystems).

### Design of a PCR strategy to selective amplify the altered spliced transcript

Specific primers were designed to selectively amplify the transcript with the altered splice pattern from patient cDNA (Figure 1C). We used a forward primer partially overlapping the exon-intron boundary (Figure 1C) (Ver-CHD7-F 5′-CAA​AGA​TGA​AAT​AGA​TGT​ATG-3′) and a reverse primer in the next exon (Ver-CHD7-R 5' TGTGGCATGTATTTCCATGG 3') to amplify a small fragment, which was then Sanger sequenced (Figure 1C).

## Results

### *CHD7* analysis in patients

The heterozygous c.5607+17A>G variant in *CHD7* intron 27, identified in two unrelated CHARGE syndrome patients, is absent from genomic and disease-related databases: GnomAD (https://gnomad.broadinstitute.org), ClinVar and LOVD (Leiden Variation Database https://databases.lovd.nl/). Varsome’s automated classification (https://varsome.com) assigns this variant as a VUS (ACMG class 3, and by the latest version as LB).

MLPA analysis ruled out intragenic or whole *CHD7* gene deletions that are known to occur in a small proportion of CHARGE patients (6%).

The c.5607+17A>G variant was evaluated with software that predicts the impact of genetic variants on splicing: VarSeak (https://varseak.bio), SpliceAid (http://www.introni.it/splicing.html) and Alternative Splice Site Predictor (ASSP, http://wangcomputing.com/assp/index.html).

### Bioinformatic prediction of c.5607+17A>G variant effects

The effect of several nucleotide substitutions at position c.5607+17 was analyzed with the VarSeak prediction software. The A>G transition found in our patients, at position + 17 was classified as a benign class 1 variant, however its presence results in the emergence of a class 3 score at position +13. Interestingly, substituting wt adenine with thymidine (A>T) or with cytosine (A>C), mimicking transversions at the same nucleotide position, were classified as benign class 1 variants (Supplementary Figure S1).

### The intronic variant c.5607+17A>G IVS27 alters splicing

pSPL3, bearing an artificial exon-intron-exon structure and a multiple cloning site, was used as an exon trapping vector. The insertion of wt or variant genomic fragments generated synthetic minigenes that were able to detect splicing alterations.

Each construct was transfected into HEK293 cell lines and, after 72 h, total RNA was extracted and retro-transcribed with random hexamers to generate cDNA. Sequence analysis of specific PCR fragments spanning the 3′-end of exon 27 and the 5′ end of exon V2 showed the expected correctly spliced transcript from the wt construct (Figure 1B); on the other hand, the construct bearing the A>G variant yielded two partially overlapping sequences indicative of two splicing products. To aid interpretation, the alleles were isolated by cloning. The correctly spliced transcript was detected, together with an aberrant transcript retaining 12 bases from the 5′ end of intron 27 spliced to the acceptor site of exon V2. Thus, our data strongly suggest that the A>G variant unmasks a cryptic donor splice site four bases upstream of its position (Figure 1B). Interestingly, the extra 12 bp open reading frame introduced by the cryptic splice site causes premature protein termination due to introduction of a STOP codon in the second triplet (Figure 1B). cDNA cloning of the mutant minigene also showed that the cryptic splice site is preferred over the physiological donor splice site: in fact, 72% of analyzed clones (*n* = 20) bore the abnormal cDNA.

### PCR amplification of pathologically spliced allele

In order to confirm the splicing pattern observed with the minigene derived from the patient’s cDNA, a PCR strategy was designed to specifically amplify the pathologically spliced allele. Using the residual RNA obtained from peripheral lymphocytes of one patient, a small amount of cDNA was generated to specifically amplify the region where intron retention occurs, based on the results of the minigene assay.

In order to exclusively amplify altered cDNA, the forward primer was designed to span the exon-intron 27 junction, while the reverse primer was designed on exon 28. Sequencing the fragment verified the aberrant transcript predicted by the minigene, confirming the pathogenetic role of the identified variant (Figure 1C).

### The a to G transition of the c.5607+17A>G IVS27 intronic variant altering splicing is specific

We used additional software, ASSP (http://wangcomputing.com/assp/index.html), to test the effect of nucleotide changes at position c.5607 Generation of a cryptic donor splicing site at base +13, was predicted only for the A>G substitution, confirming the *in vitro* data obtained so far (Supplementary Figure S2).

Our results were then corroborated using the minigene constructs to study the effects of A>C and A>T transversions on the splicing mechanism at position c.5607+17. In line with VarSeak and ASSP predictions, these substitutions did not result in any detectable abnormal splicing.

The sequences from the wt, mutant and synthetic variants inserts were analyzed using SpliceAid software (Piva et al., 2009). The substitution of A>G was predicted to create a recognition site for the ETR3 (CELF-2) splicing regulatory protein (window of four to six bases, UAUGU sequence) (Dujardin et al., 2014) (Supplementary Figure S1). While the A>C substitution did not generate any specific RNA recognition site, the A>T “synthetic” substitution generated a sequence motif (AUUUUG) recognized by TIA-1 (T-cell-restricted intracellular antigen 1) and TIAL1 factors (TIA1-like 1) with a low score of two out of 10 with SpliceAid, and 5 with SpliceAid2. The expression of ETR3 in HEK293 cells was confirmed by PCR on cDNA (Supplementary Figure S3).

## Discussion

The vast amount of data generated by new sequencing technologies does not always allow the nature of an identified variant to be conclusively defined. This is particularly critical for conditions presenting with high clinical heterogeneity. Some variants, such as intronic variants, are by their own nature more difficult to interpret accurately. Despite the use of *in silico* programs to predict the functional impact of intronic sequence variations, experimental confirmation is always preferred.

CHARGE syndrome is a rare and highly heterogeneous condition. Patients do not always present with all of the common features, and often have additional findings. Furthermore, the cardinal features of CHARGE syndrome are seen in other conditions, adding to the diagnostic challenges. The clinical variability is also present within families, as sibpairs often show distinct features (Jongmans et al., 2006). In line with previous reports of CHARGE syndrome variability, the two patients included in this study, albeit sharing the same variant in *CHD7*, present with different clinical pictures: the first patient has two major (choanal atresia and agenesis of the semicircular canals) and one minor criteria (hypogonadism) according to Verloes (2005). The second patient shows one major (bilateral coloboma) and several minor criteria. Both patients do not satisfy all the criteria for typical CHARGE syndrome and would be classified as Atypical or Partial cases of the Syndrome (Verloes, 2005). The occurrence of atypical phenotypes associated with pathogenic *CHD7* variants is frequent and well documented (Hale et al., 2016; Legendre et al., 2017; Thomas et al., 2022). The cause of such high variability seen in CHARGE syndrome is unknown; it is most likely that differences in the individual genetic backgrounds are sufficient to modulate phenotypic expression, but this remains an area for future studies.

In our study, we characterized c.5607+17A>G, a novel *CHD7* variant located in intron 27, found in two unrelated patients with CHARGE syndrome.

We believe that this variant is pathogenic because:

1) it has not been previously reported in genome databases of apparently healthy individuals;
2) adenine at this position is fairly conserved among different species (Supplementary Figure S4);
3) the same variant was identified in two unrelated patients with CHARGE syndrome, and it occurs *de novo* in the one patient tested;
4) the mutant minigene construct shows aberrant splicing;
5) altered splicing was detected in the cDNA synthesized from RNA of one of our patients.

Bioinformatic analysis of c.5607+17A>G with splicing prediction software was not sufficient to establish a pathogenetic effect of this variant, and a very limited amount of cDNA was available, from only one of the patients. There are reports of patients with splice site mutations within *CHD7* (Jongmans et al., 2006). The clinical presentation in these patients appear to show the same clinical features and the extent of the variability with the rest of the cohort. Due to these limitations, we decided to use a minigene assay approach to characterize this variant.

Minigenes carrying intron and exon regions with variant and wt nucleotides were generated. Analyzing cDNA from transfected cells clearly demonstrated an effect of the variant on splicing, giving rise to aberrant RNA and resulting in a premature stop codon. Furthermore, this result was confirmed on the residual amount of cDNA available from one of our two patients.

To gain further evidence of a pathogenetic effect, the specificity of the adenine to guanidine substitution was assessed bioinformatically, by also evaluating the impact of cytosine and thymidine substitutions at the same position with VarSeak software. The resulting score clearly indicates a benign effect of the two transversions (Supplementary Figure S2). Two additional minigenes bearing these substitutions (A>C and A>T) were generated to test this prediction. Expression of these constructs in cultured cells showed no aberrant splicing, confirming *in silico* results.

Using an additional software SpliceAid (Piva et al., 2009), we tried to understand the pathogenetic mechanism of the A>G substitution: compared with the wt sequence of intron 27, the G variant creates a consensus sequence (a window of four to six bases) specific for the binding protein ETR-3 (CELF-2), a member of the CELF family of splicing regulators (Supplementary Figure S1). The prediction reached a significant maximum value of nine out of 10 with SpliceAid (UAUGU sequence), and five for each window with SpliceAid2 (Piva et al., 2009). ETR-3/CELF-2 is a component of the eukaryotic splicing machinery (Dujardin et al., 2014), and has been studied for its role in alternative splicing (Charlet-B et al., 2002) and post-transcriptional regulation of neural stem cell fate (MacPherson et al., 2021). It is widely expressed, particularly in the brain, heart and muscle tissues (Ladd et al., 2001). Mutations in ETR-3/CELF-2 are also associated with an autosomal dominant form of developmental and epileptic encephalopathy (DEE97, MIM 619561). Interestingly, silencing of ETR-3/CELF-2 expression has been tested in a mouse model of spinal and bulbar muscular atrophy (SBMA) as a strategy to modulate the phenotype of this condition (Miyazaki et al., 2012). Although ETR-3/CELF-2 cannot at present be linked mechanistically with the variant identified in the present study, it is tempting to speculate a causative link. Nevertheless, ETR-3 expression in HEK293 cell lines used in this study was confirmed by cDNA amplification (Supplementary Figure S3).

The comparison of the wt sequence with the “synthetic” A>C variant with the same software, did not identify any exclusive recognition sequences for splicing proteins. In contrast, the A>T “synthetic” substitution generated a sequence motif (AUUUUG) recognized by TIA-1 (T-cell-restricted intracellular antigen 1) and TIAL1 factors (TIA1-like 1, referred also as TIAR), but with a very low score of two out of 10 with SpliceAid, and 5 with SpliceAid2. TIA1 and TIA1L are RNA recognition motif proteins (Dember et al., 1996), ubiquitously expressed and playing an important role in several aspects of mRNA maturation, translation, and RNA-stress-sensing pathways in human cells (Meyer et al., 2018). However, the very low SpliceAid score and the absence of aberrant cDNAs *in vitro*, suggest that any potential aberrant splicing due to an A>T substitution occurs at best at a very low rate and below a pathological level. On the other hand, additional analysis of the A>G substitution, confirmed the activation of the cryptic splice site at position +12 (score 5.379), in line with our wet lab experiments. It is notable that not only the position, but also the specific base substitution, seems to be critical for a pathological effect.

Determining the impact of an unknown variant on splicing can be achieved by analyzing the patient’s RNA. RNA sequencing, for instance, could be informative in these cases, and should, in principle, be able to detect alterations in the expression or splicing of any gene. However, patient material is not always available for analysis, and it may not match the tissue specificity of the disease. Without a source of RNA from the individual that carries the variant, there are few tools that can be implemented. Exon trapping vectors, such as used in this study, allow investigators to determine whether a variant alters RNA splicing, by expressing reference and variant minigenes in mammalian cells and analyzing the resulting RNA products (Wang and Marín, 2006; Tompson and Young, 2017). Many examples have confirmed the broad validity of this approach to study the impact of intronic variants in *BRCA1* (Steffensen et al., 2014), in *MLH1*, *MSH2* and *MSH6* for colorectal cancer (Petersen et al., 2013) and more generally to study mRNA splicing (Basei et al., 2021). In this kind of experiments, different cell types have been employed. In our case we used HEK293, a human derived cell line, very easy to grow and maintain. HEK293 cells are widely used in several laboratory applications including minigene assays. For instance, it has been used by Van der Klift and others to prove the effects of several mutations in Lynch syndrome finding a high correlation concordance between minigene assays and patient RNA analyses (Van der Klift et al., 2015). In another work, Yin and collaborators used the minigene assay to verify the effect of an intronic variant in the SCN5A gene causing a form of incessant ventricular tachycardias (Yin et al., 2021). These results demonstrate that HEK293 is a reliable cell line to perform minigene assay.

There are cases were this approach hits its own technical limitations. For instance, it can be difficult to generate a minigene for deep intronic variants. The analysis of such variants would require the construction of huge vectors that can be difficult to generate and to transfect. A rationalized design of a vector could bypass the need for the entire genomic sequence, but it could add more variability. One way to overcome such limitations would be to introduce the desired point mutation with a Gene Editing approach in a suitable cell line and to compare the effect of the studied variant on the expression of the gene of interest.

Systematic implementation of these approaches in the clinical setting is not straightforward since many resources need to be allocated. Nevertheless, they represent a powerful tool to determine the diagnostic relevance of VUS, and further technological advances are desirable to make them compatible with the clinical workflow.

## Data Availability

The datasets for this article are not publicly available due to concerns regarding participant/patient anonymity. Requests to access the datasets should be directed to the corresponding author.
