# Supplementary figures and images for: Case report: Functional characterization of a novel CHD7 intronic variant in patients with CHARGE syndrome
Source: Front Genet. 2023 Feb 9;14:1082100. doi: 10.3389/fgene.2023.1082100 (PMC9947648; doi:10.3389/fgene.2023.1082100)

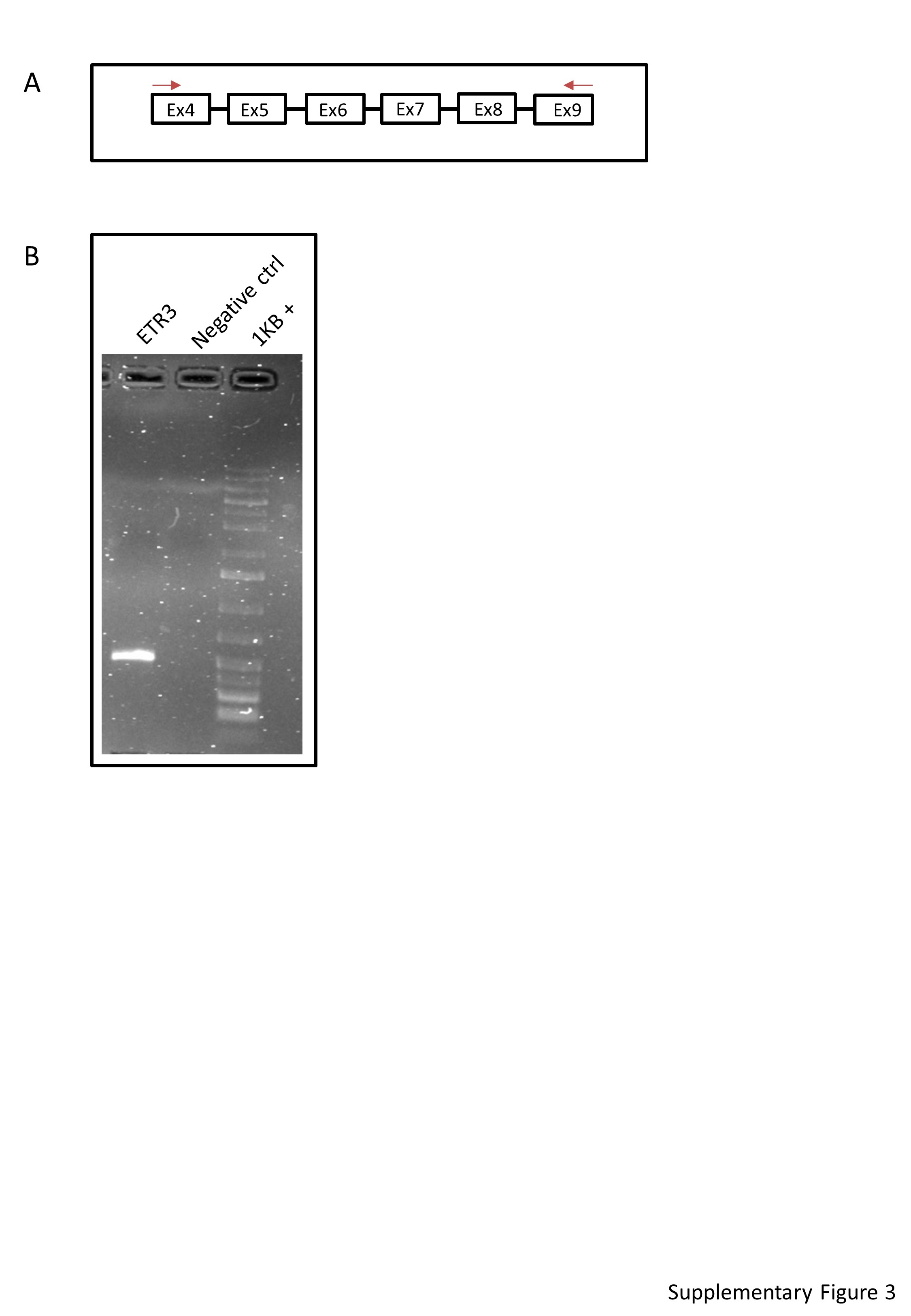

Supplement: Supplementary file 1 [file Image3.jpg]

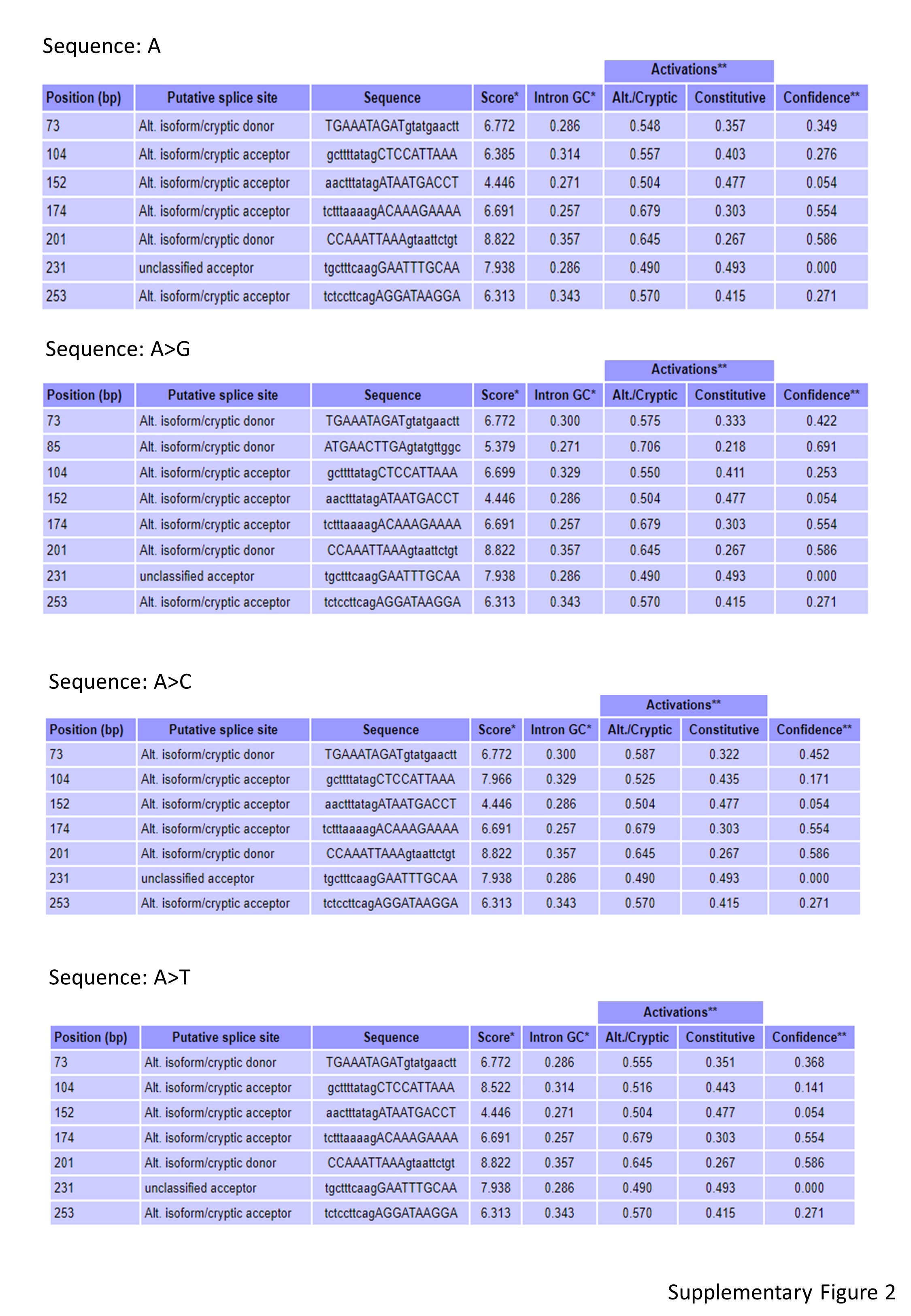

Supplement: Supplementary file 2 [file Image2.jpg]

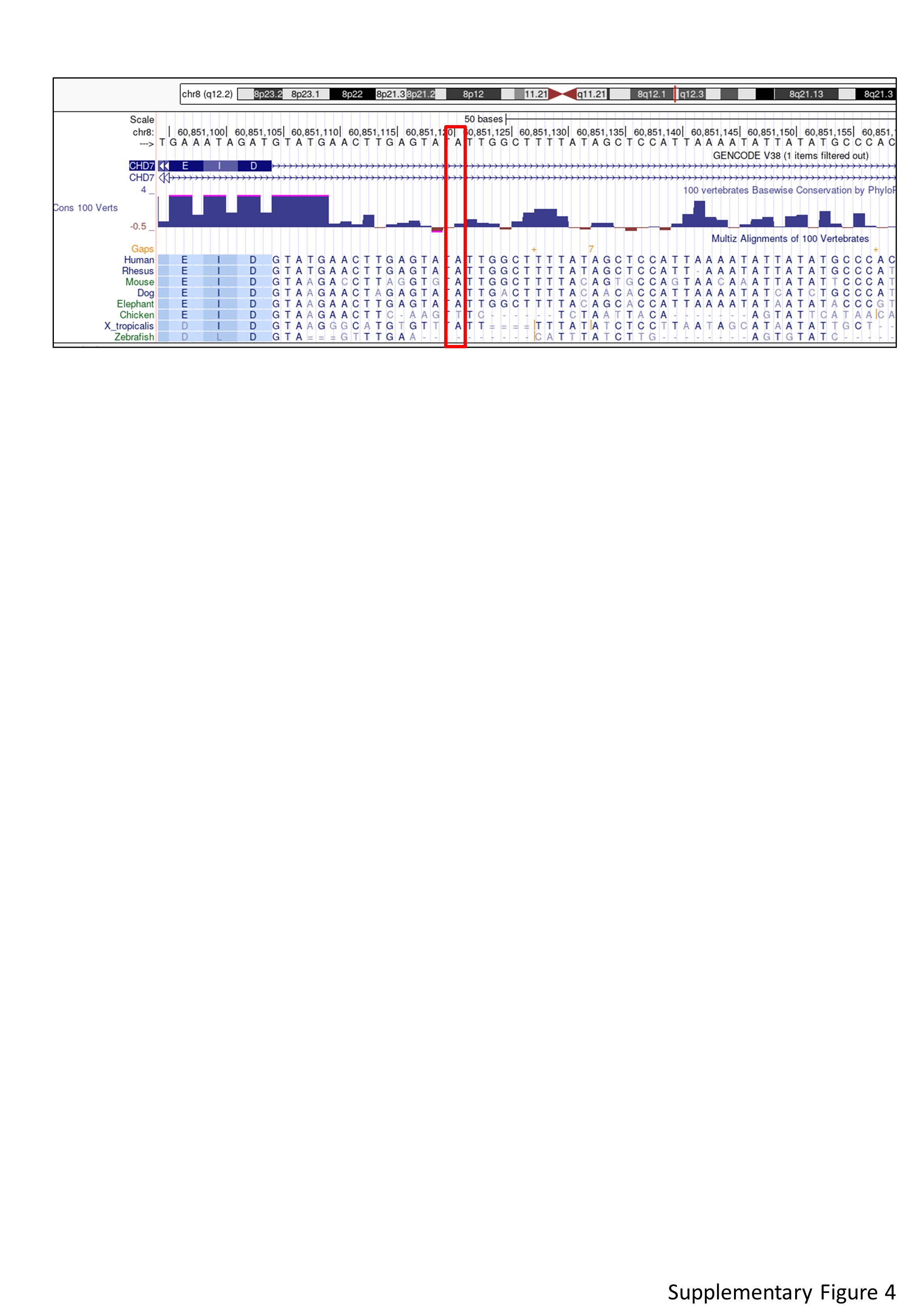

Supplement: Supplementary file 4 [file Image4.jpg]

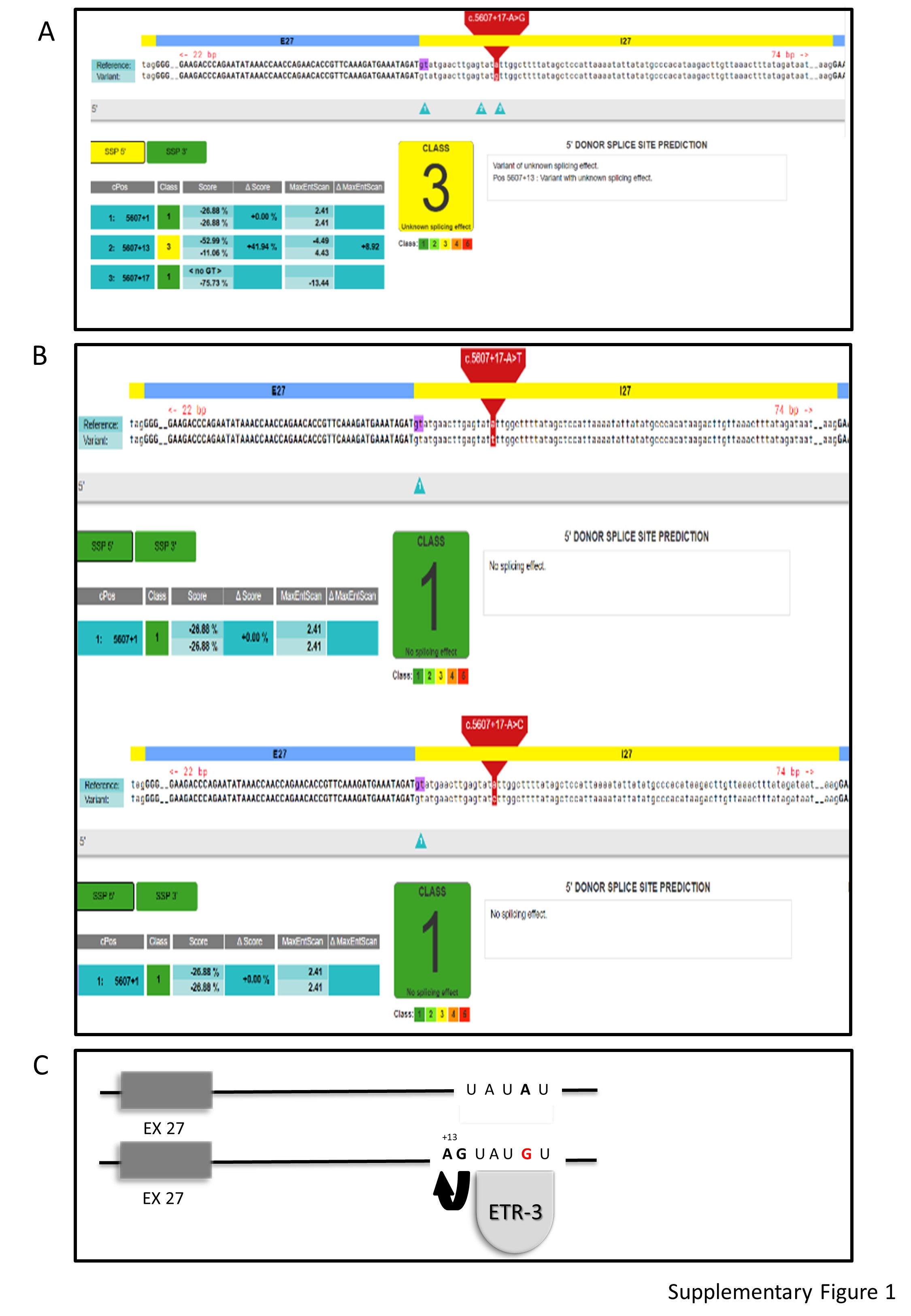

Supplement: Supplementary file 5 [file Image1.jpg]
