# Supplementary material for: Case report: Functional characterization of a novel CHD7 intronic variant in patients with CHARGE syndrome
Source: Front Genet. 2023 Feb 9;14:1082100. doi: 10.3389/fgene.2023.1082100 (PMC9947648; doi:10.3389/fgene.2023.1082100)
Supplement: Supplementary file 3 [file Presentation1.pdf]

**Supplementary Material**

# Functional characterization of a novel *CHD7* intronic variant in patients with CHARGE syndrome

Cesare Rossi[1], Sherin Ramadan[2], Cecilia Evangelisti[1,2], Simona Ferrari[1], Maria Accadia[3], Reha M. Toydemir[4], Emanuele Panza[1,2]

[1]UO Genetica Medica, IRCCS Azienda Ospedaliero-Universitaria di Bologna, Italia;
[2]Dipartimento di Scienze Mediche e Chirurgiche, Università di Bologna, Italia;
[3]Servizio di Genetica Medica, Ospedale "Cardinale G. Panico", Tricase (LE), Italia;
[4]Department of Pediatrics, University of Utah, Salt Lake City, Utah, USA.

## Extended description of minigene construction

An *in vitro* splicing assay was developed, by amplifying a genomic region including intron 26, exon 27 and intron 27 of *CHD7* from a control genomic DNA. The oligonucleotides used to amplify this fragment contained restriction sites for XhoI and BamHI, necessary for subsequent cloning steps. The fragment was amplified with PrimeSTAR GXL DNA Polymerase (Takara), cloned in TOPO ZERO blunt cloning vector and 2 µl transformed into Top10 competent cells provided with the kit, following manufacturer's recommendations (Thermo Fischer).

Different nucleotide substitutions were introduced by site directed mutagenesis with an in-house protocol. Briefly, complementary and overlapping 46 nt long primers bearing the desired nucleotide changes were designed (sequence of primers available upon request). These primers were used to anneal to plasmid DNA in an "around the world" PCR, using a high fidelity Taq polymerase (Phusion polymerase, Thermo Fischer). At the end of the reaction, the PCR product was digested with one unit of DpnI for one hour at 37°C, and 5 µl was transformed into chemical competent DH5α competent cells. The plasmids containing the wt and the variant fragments were verified by Sanger sequencing (3730 Instrument, Applied Biosystems).

The pZERO plasmids, and the pSPL3 exon trapping vector were digested using XhoI and BamHI restriction enzymes (Thermo Fischer) and fragments were extracted from the gel, following manufacturer's procedures (Combo kit, Gene All).

Ligation of inserts into the pSPL3 vector was carried out by incubating them for 2 hours at room temperature with T4 DNA ligase (Thermo Fischer) followed by transformation of DH5α

chemical competent bacteria. The clones obtained were verified by PCR and positive clones were expanded in liquid medium (Lennox medium, Thermo Fischer) with the appropriate antibiotic. The next day, plasmid DNA was extracted and purified (Plasmid Purification kit, Gene All). Clone sequences of pSPL3_CHD7-WT and pSPL3_CHD7-MutG, pSPL3_CHD7-SyntT, and pSPL3_CHD7-SyntC, were all confirmed by direct sequencing (3730 Instrument, Applied Biosystems). pSPL3 vectors were expanded and purified by column extraction using Midi columns (Qiagen, Midi kit), for preparation of electroporation grade plasmid DNA. Primer sequences are available upon request.

## WEB RESOURCES

VarSeak: https://varseak.bio

GnomeAD: https://gnomad.broadinstitute.org

Ensembl: https://www.ensembl.org/index.html

SpliceAid: http://www.introni.it/splicing.html

ASSP: http://wangcomputing.com/assp/index.html

**Supplementary Figure 1.**

A) VarSeak prediction of A>G variant effect. B) VarSeak prediction of A>T and A>C transversions. C) Schematic representation of the generated recognition sequence for ETR-3/CELF-2, and position of the predicted newly activated splice site.

**Supplementary Figure 2.**

Analysis with ASSP software showing the effect of the A>G substitution compared to wt and synthetic substitutions. Only the A>G substitution generates a cryptic donor splicing site at the expected position based on minigene assay experiments.

**Supplementary Figure 3.**

A) Primer design for ETR-3/CELF-2 exons. B) Amplification of ETR-3/CELF-2 cDNA obtained from HEK293 RNA, showing expression in the cell line used in this study, along with a PCR negative control.

**Supplementary Figure 4.**

UCSC genome browser capture showing that adenine at position 5607+17 is fairly conserved among different species.
